# Dietary Coated Sodium Butyrate Ameliorates Hepatic Lipid Accumulation and Inflammation via Enhancing Antioxidative Function in Post-Peaking Laying Hens

**DOI:** 10.3390/metabo13050650

**Published:** 2023-05-10

**Authors:** Sasa Miao, Yan Li, Tianming Mu, Xiaoming Wang, Wenyan Zhao, Ru Li, Xinyang Dong, Xiaoting Zou

**Affiliations:** Key Laboratory of Animal Feed and Nutrition of Zhejiang Province, Key Laboratory of Animal Nutrition and Feed Science (Eastern of China), Ministry of Agriculture and Rural Affairs, The Key Laboratory of Molecular Animal Nutrition, Ministry of Education, College of Animal Sciences, Zhejiang University, Hangzhou 310058, China

**Keywords:** coated sodium butyrate, lipid metabolism, oxidative stress, antioxidative function, laying hen

## Abstract

During the aging process of laying hens, hepatic oxidative stress damage and lipid accumulation are prone to occur, leading to the deterioration of egg quality and a decline in production properties. This research was designed to explore the effects of different levels of coated sodium butyrate (CSB) addition on oxidation resistance, inflammatory reaction, lipid metabolism and hepatic oxidative damage-related gene expression in aged laying hens. A total of 720 healthy 52 weeks old Huafeng laying hens were arbitrarily divided into 5 groups of 6 replicates with 24 birds each and fed a basal diet supplemented with 0, 250, 500, 750 and 1000 mg/kg CSB for 8 weeks, respectively. The CSB quadratically upgraded GSH-Px activities and downgraded MDA content in the liver and serum. The LDL-C, NEFA and TG contents decreased quadratically in CSB groups and significantly reduced the fatty vacuoles as well as the formation of fat granules in the liver (*p* < 0.05). Meanwhile, the CSB quadratically upregulated the gene expression of IL-10, Nrf2 and HO1, but downregulated the gene expression of IFN-γ, TNF-α and Keap1 in a quadratic manner (*p* < 0.05). Moreover, the CSB quadratically degraded the mRNA level of fatty acid synthesis but increased the gene level of key enzymes of fatty acid catabolism (*p* < 0.05). In conclusion, dietary CSB supplementation has a favorable effect in protecting against liver injury and alleviating lipid accumulation and inflammation by enhancing hepatic antioxidative function in aged laying hens.

## 1. Introduction

Modern intensively produced laying hens are vulnerable to the challenges of inflammatory reactions, fat deposition, autophagy and hepatic oxidative stress after they enter the peak laying stage [1]. The occurrence of this phenomenon causes a decline in production performance and deterioration of egg quality, which seriously affects the economic efficiency of the poultry industry [2,3]. It is well known that the liver, as an important organ of lipid metabolism in birds, is involved in the new synthesis and transport of lipids [1]. However, during the aging process of hens, excessive lipid accumulation not only leads to hepatocyte death but also gradually generates oxidative stress damage, leading to a decline in liver antioxidant capacity, liver dysfunction and liver steatosis [4,5,6]. Under normal conditions, the body’s antioxidant system protects the body from oxidative damage, especially antioxidant enzymes such as superoxide dismutase (SOD), catalase (CAT) and glutathione peroxidase (GSH-Px), which minimize oxidative stress in cellular organelles [7]. In addition, genetic, nutritional, hormonal and environmental factors can also cause disturbances in hepatic lipid metabolism and oxidative stress in chickens [4]. Although metabolic diseases in laying hens are multifactorial, nutritional factors are key to the etiology of hepatic lipid metabolism [8]. Therefore, nutritional regulation to assess the antioxidant capacity and liver lipid metabolism in older laying hens is essential to prevent chicken disease and maintain health.

Butyric acid, which is mainly derived from the fermentation of dietary fiber by intestinal bacteria, is an important organic acid additive in the animal body with similar antibiotic disease prevention and growth promotion effects and is safe with no residue [9]. Sodium butyrate (SB), which can be converted to butyric acid in the digestive tract of birds, has attracted widespread attention for practical production [9,10,11]. Recently, accumulating studies have found that SB can alleviate metabolic diseases through its anti-inflammatory and antioxidant effects, and reducing oxidative stress and pathogenic microbiota in the intestine [10,12,13,14]. A study on chickens indicated that dietary SB addition could relieve the oxidative damage caused by corticosterone exposure and decrease malondialdehyde (MDA) levels while increasing SOD activity [12,15]. Nevertheless, SB has an offensive odor and pungent taste, which can have a negative impact on animal intake, and easily absorbs moisture when exposed to air [11]. CSB has no negative effect on feed intake and could increase feed efficiency [16]. Therefore, most of the SB products currently used in practice are fat- or starch-coated to reduce the pungent odor of the butyrate itself and to provide a slow release in the intestinal tract with a more stable effect than uncoated SB [11,17].

Recent studies have demonstrated that SB plays a vital role in influencing production performance, enhancing meat quality, improving gut immunity and regulating the intestinal microbiota of animals [14,18,19]. Nevertheless, to our knowledge, there are no available data to assess the effects of CSB on the hepatic health status and lipid metabolism of post-peak laying hens. Late laying hens are prone to lipid deposition, inducing diseases related to lipid metabolism and accompanied by symptoms such as fatty liver, inflammation and bleeding of the liver [20]. Moreover, oxidative damage, abnormal hormone secretion and reduced immunosuppression were found in the late laying period of laying hens [21]. Thus, our aim was to explore the underlying mechanism of liver oxidative injury during the aging process of post-peak laying hens and the protective effects of CSB by reducing the accumulation of lipids and enhancing the antioxidant properties of the liver. Moreover, the relationship between CSB and the association of endoplasmic reticulum stress (ERS) indexes, antioxidant parameters, inflammatory reactions and hepatic lipid metabolism was also assessed.

## 2. Materials and Methods

### 2.1. Experimental Design, Animals and Diet

The experimental protocols used in this study were approved by the Animal Care and Welfare Committee of Zhejiang University (No. ZJU2013105002) (Hangzhou, China).

After one week of acclimatization, 720 healthy 52 weeks old Huafeng laying hens, whose initial egg production rate was at 73.60 ± 0.27%, were arbitrarily assigned to 5 groups with 6 replicates, namely, the control group (basal diet), S250 group (basal diet +250 CSB mg/kg), S500 group (basal diet +500 CSB mg/kg), S750 group (basal diet +750 CSB mg/kg) and S1000 group (basal diet +1000 CSB mg/kg). CSB (sodium butyrate content was 50%, coated with palm oil and silica) was purchased from Hangzhou Dade Biotechnology Co., Ltd. (Hangzhou, China). The test period lasted for 8 weeks. The hens were fed and watered freely, the coop was disinfected regularly, the ventilation and lighting conditions were identical, and the average daily light was 16 h. The basal diet composition is shown in Table 1.

### 2.2. Sample Collection

After the experiment, 12 hens in each group (2 hens per repetition) were arbitrarily selected to fast for 12 h. Blood samples were collected from wing veins, separated by centrifugation (3000 rpm/10 min) and immediately stored at −80 °C. The hens were then slaughtered, and the liver tissue was fixed with 4% paraformaldehyde or wrapped in tin foil and frozen at −80 °C for subsequent detection.

### 2.3. Assessment of Liver Injury

To visualize the extent of liver damage, the paraformaldehyde-fixed liver samples were paraffin-embedded for Hematoxylin and Eosin (H&E) staining. Blocks of liver tissue were cut and snap-frozen in liquid nitrogen, and frozen sections were embedded and used for Oil Red O (ORO) staining. Then, liver tissue injury was observed using a panoramic scan of the pathological sections.

### 2.4. Liver Lipid Profile

The levels of total cholesterol (TC), triglyceride (TG), high-density lipoprotein cholesterol (HDL-C) and low-density lipoprotein cholesterol (LDL-C) levels were determined using an automatic biochemical analyzer. Non-esterified fatty acids (NEFA) were detected using the NEFA-ELISA commercial kit according to the instruction manual.

### 2.5. Determination of Antioxidant Capacity

Approximately 0.5 g of liver tissues from each repetition were homogenized with 4.5 mL of hypothermic PBS, and then the homogenates were centrifuged at 3500 r/min at 4 °C for 15 min to obtain 10% liver tissue homogenate. The protein content of the samples was determined using the BCA protein assay kit (AR1189, Boost Biotechnology Co., Ltd., Wuhan, China). Serum and liver antioxidant status-related indicators, such as CAT, SOD, MDA, GSH-Px and total antioxidative capacity (T-AOC), were measured and calculated according to the kit procedure (Nanjing Jiancheng Bioengineering Institute, Nanjing, China).

### 2.6. Quantitative Real-Time PCR (qRT-PCR) Analysis

Quantification and reverse transcription of mRNA were the same as described previously [22,23]. A total of 10–20 mg of fresh liver tissue was placed into 1 mL of Trizol reagent (TransGen Biotech Co., Ltd., Beijing, China) to extract the total RNA. After determining the purity and concentration using a NanoPhotometer (N60, IMPLEN, München, Germany), 1 μg of total RNA was reverse-transcribed into complementary DNA according to the kit instructions. RT-PCR reactions were then performed using the Applied Biosystems Quant Studio 3 Real-Time PCR System and the SYBR Premix Ex TaqTM kit. The reaction conditions were as follows: 95 °C for 3 min, 95 °C for 10 s, 60 °C for 40 s, and 40 cycles. The primer sequences for the forward and reverse primers are listed in Table 2. The β-Actin was used as an internal reference gene. The differential expression was calculated using the 2^−ΔΔCt^ method [24].

### 2.7. Statistical Analysis

The data were subjected to one-way ANOVA using SPSS 20.0 (SPSS Inc., Chicago, IL, USA) and expressed as mean ± SEM. Treatment means were separated using the Tukey least significant difference post hoc test at the *p* < 0.05 statistical level. When significant differences were found, orthogonal polynomial contrasts were further used to examine the linear and quadratic effects of the different inclusion levels of dietary CSB. In addition, Spearman’s correlation analysis was used to explore the relationship between antioxidant indices and other key parameters. Cluster correlation heat maps and networks with signs were performed using the OmicStudio tool at https://www.omicstudio.cn.

## 3. Results

### 3.1. CSB Enhanced Serum and Hepatic Antioxidant Properties

**Figure 1:**
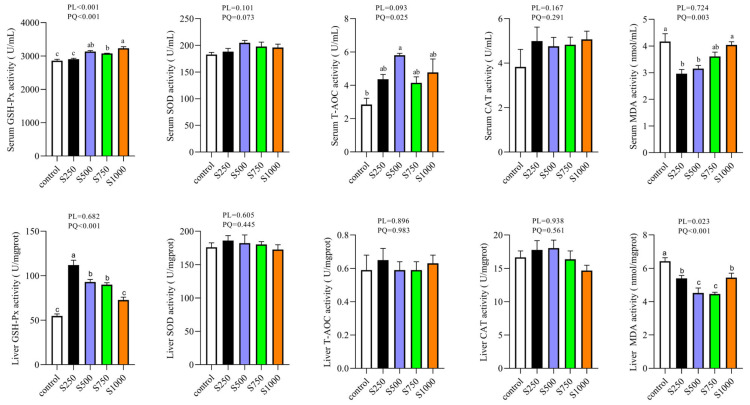
Effect of CSB supplementation on antioxidant capacity of serum and liver of laying hens (*n* = 6). PL: *p*-value for linear effect. PQ: *p*-value for quadratic effect. ^a–c^ Means with unlike letters are significantly different (Turkey, *p* < 0.05).

The antioxidant indexes in the liver tissue and serum are shown in Figure 1. GSH-Px activities in the liver tissue and serum were quadratically elevated under CSB influence (*p* < 0.05). T-AOC activity only in the serum was upregulated in a quadratic manner (*p* < 0.05). MDA content was quadratically lower in both the serum and liver with the dietary CSB addition (*p* < 0.05).

### 3.2. CSB Decreased Liver Lipid Droplets

**Figure 2:**
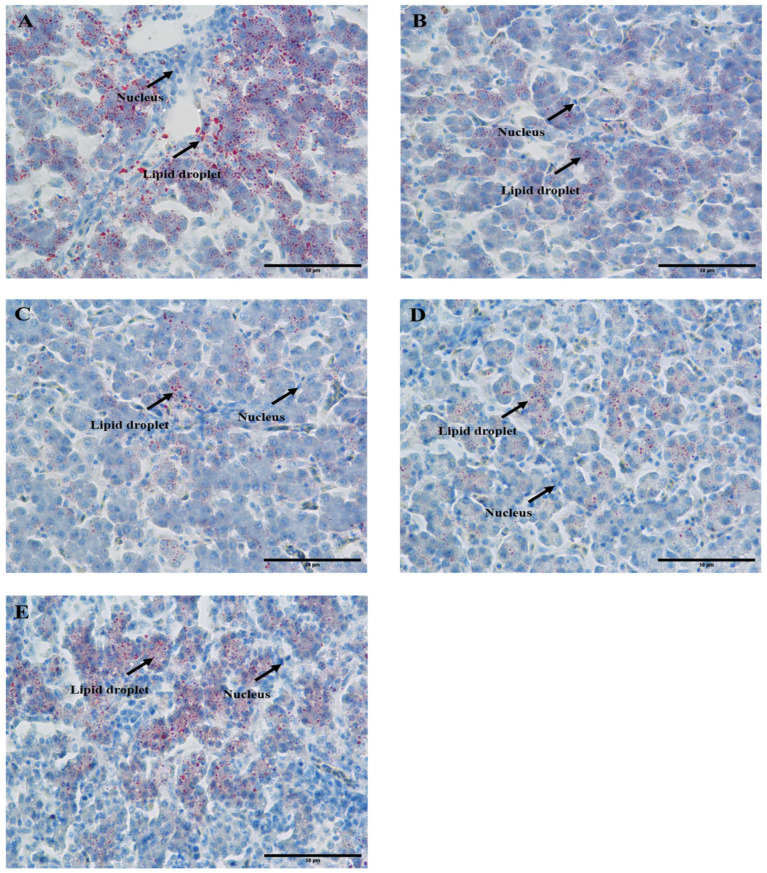
Hepatic lipid metabolism response to dietary CSB treatment in post-peak laying hens (*n* = 6). Liver Oil red o staining (scale: 50 μm). (**A**) Control; (**B**) S250; (**C**) S500; (**D**) S750; (**E**) S1000.

To explore whether CSB could lessen the lipid deposition in the liver, the liver was subjected to Oil Red O staining. The result is displayed in Figure 2. The formation of liver lipid droplets was significantly reduced in the S250, S500 and S750 groups with the increase in CSB addition. Nevertheless, this decreasing trend was markedly attenuated in the S1000 group.

### 3.3. CSB Regulated Hepatic Lipid Profile

**Figure 3:**
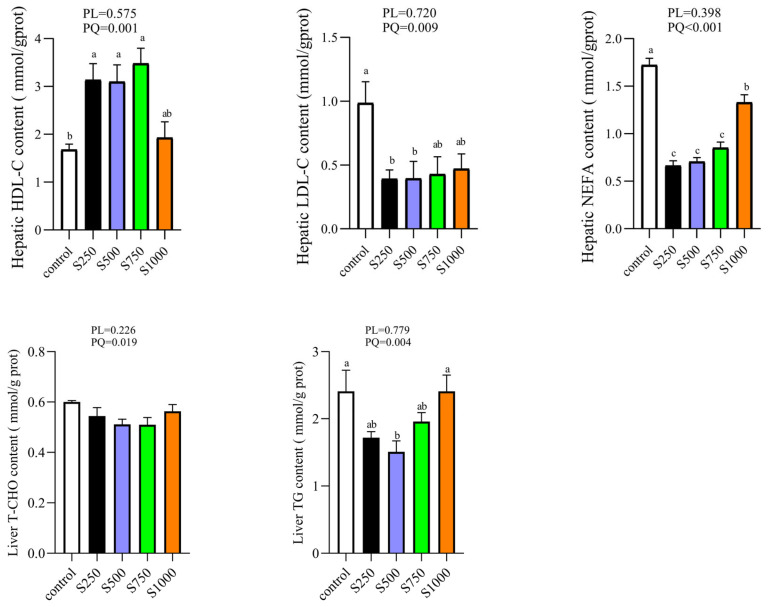
Effect of CSB supplementation on lipid profile in the liver of laying hens (*n* = 6). PL: *p*-value for linear effect. PQ: *p*-value for quadratic effect. ^a–c^ Means with unlike letters are significantly different (Turkey, *p* < 0.05).

Oil Red O staining revealed that CSB could attenuate liver fat deposition. Simultaneously, our previous study demonstrated that CSB quadratically decreased the TG content in the serum [20]. Thus, we further investigated the mitigation function of CSB on the hepatic lipid profile. As displayed in Figure 3, CSB treatment quadratically suppressed the increase in LDL-C, NEFA and TG content (*p* < 0.05), whereas it elevated the hepatic HDL-C content in a quadratic manner (*p* < 0.01).

### 3.4. CSB Alleviated Hepatic Lipid Metabolism

**Figure 4:**
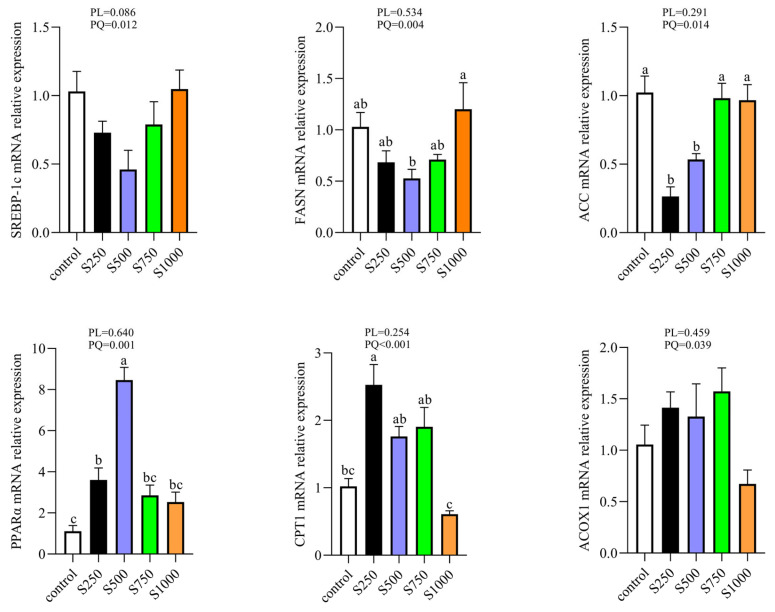
Effect of CSB supplementation on hepatic lipid metabolism in laying hens (*n* = 6). PL: *p*-value for linear effect. PQ: *p*-value for quadratic effect. ^a–c^ Means with unlike letters are significantly different (Turkey, *p* < 0.05).

To make a thorough exploration of the alleviative function of CSB on lipid metabolism, we further tested the gene expression related to lipid synthesis and fat oxidation. As exhibited in Figure 4, laying hens fed with CSB quadratically degraded the fatty acid synthesis gene expression, such as FASN and ACC (*p* < 0.05). Nevertheless, the mRNA levels of key enzymes involved in the fatty acid catabolism, including PPARα and CPT1, exhibited a quadratic increase due to the intake of CSB (*p* < 0.05).

### 3.5. Relationship between Lipid Metabolism and Liver Antioxidant Indexes

**Figure 5:**
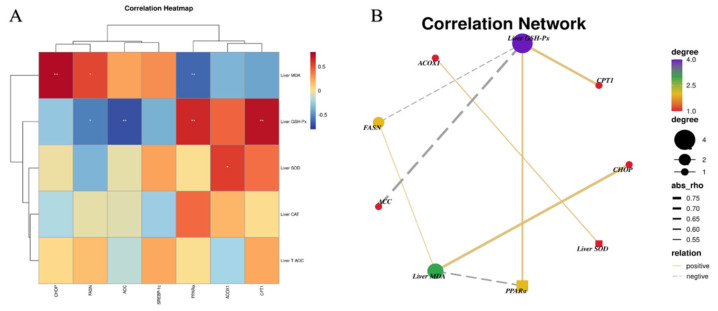
Correlation heat map (**A**) and correlation network (**B**) between lipid metabolism genes and antioxidant indexes in the liver. * *p* < 0.05 and ** *p* < 0.01.

As shown in Figure 5, the potential association between lipid metabolism−related genes and antioxidant indexes was detected in the liver. Liver GSH−Px was positively correlated with CPT1 and PPARα, but inversely correlated with ACC and FASN. Liver MDA was positively correlated with FASN and CHOP. Simultaneously, liver SOD was positively correlated with ACOX1.

### 3.6. CSB Attenuated Liver Steatosis and Inflammatory Reaction

**Figure 6:**
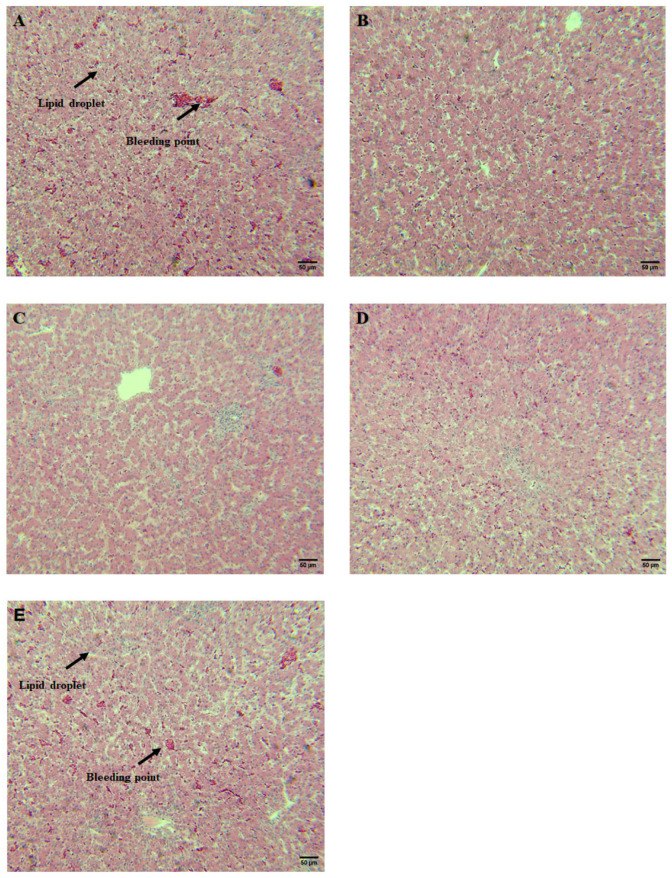
Hepatic histopathology and pathological response to dietary CSB treatment in post-peak laying hens (*n* = 6). Liver hematoxylin and eosin (H&E) staining (scale: 50 μm). (**A**) Control; (**B**) S250; (**C**) S500; (**D**) S750; (**E**) S1000.

Figure 6 displays the histopathological examination of the liver. The livers of the control group showed a large number of fatty vacuoles, disordered arrangement of hepatocytes and inflammatory cell infiltration. Overtly, hepatic fat vacuoles in CSB intervention laying hens were clearly reduced, hepatocyte arrangement returned to normal, and inflammatory cell infiltration decreased. Nevertheless, this decreasing trend was attenuated obviously in the S1000 treatment group.

### 3.7. CSB Enhanced Hepatic Anti-Inflammation Status

**Figure 7:**
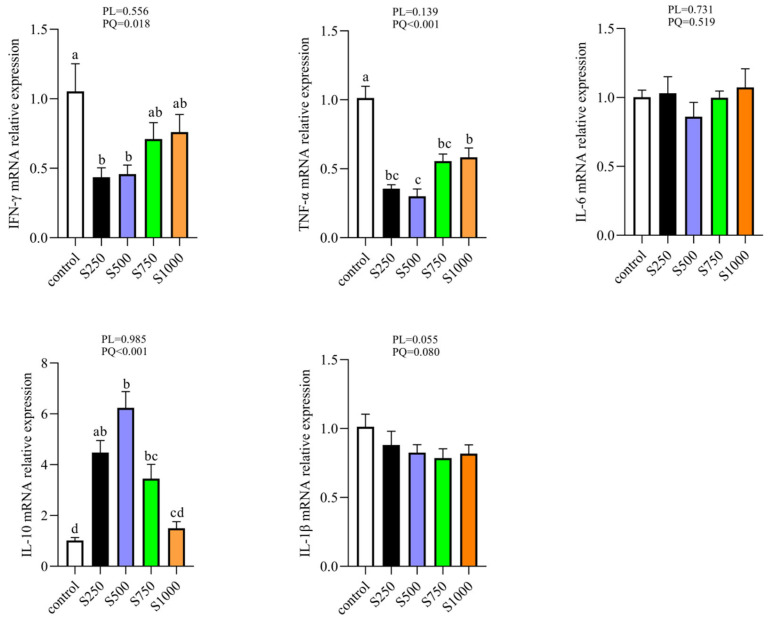
Effect of CSB supplementation on hepatic inflammatory cytokine gene expression in laying hens (*n* = 6). PL: *p*-value for linear effect. PQ: *p*-value for quadratic effect. ^a–c^ Means with unlike letters are significantly different (Turkey, *p* < 0.05).

To further examine the alleviative reaction of CSB on liver injury, we tested the hepatic inflammatory cytokines. As presented in Figure 7, the relative expression of IFN-γ and TNF-α mRNA decreased quadratically in the CSB treatment group (*p* < 0.05), whereas the IL-10 mRNA level increased in a quadratic manner in laying hens in the CSB group (*p* < 0.05).

### 3.8. CSB Regulated ERS of the Liver

**Figure 8:**
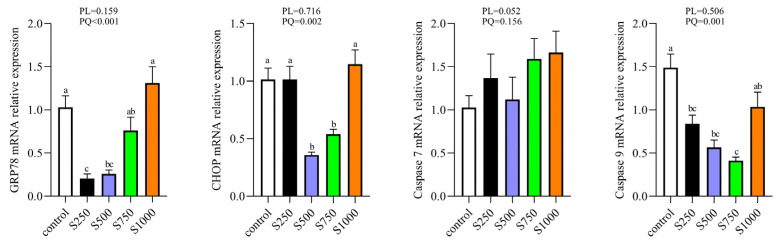
Effect of CSB supplementation on hepatic endoplasmic reticulum stress in laying hens (*n* = 6). PL: *p*-value for linear effect. PQ: *p*-value for quadratic effect. ^a–c^ Means with unlike letters are significantly different (Turkey, *p* < 0.05).

The relative expression of the levels of ER stress-related genes is shown in Figure 8. Dietary CSB addition induced the downregulation of GRP78, CHOP and Caspase 9 in a quadratic manner (*p* < 0.05), whereas no significant difference was found in the gene expression of Caspase 7 among all groups (*p* > 0.05).

### 3.9. Relationship among Hepatic Inflammatory, ERS Indexes and Antioxidant Parameters

**Figure 9:**
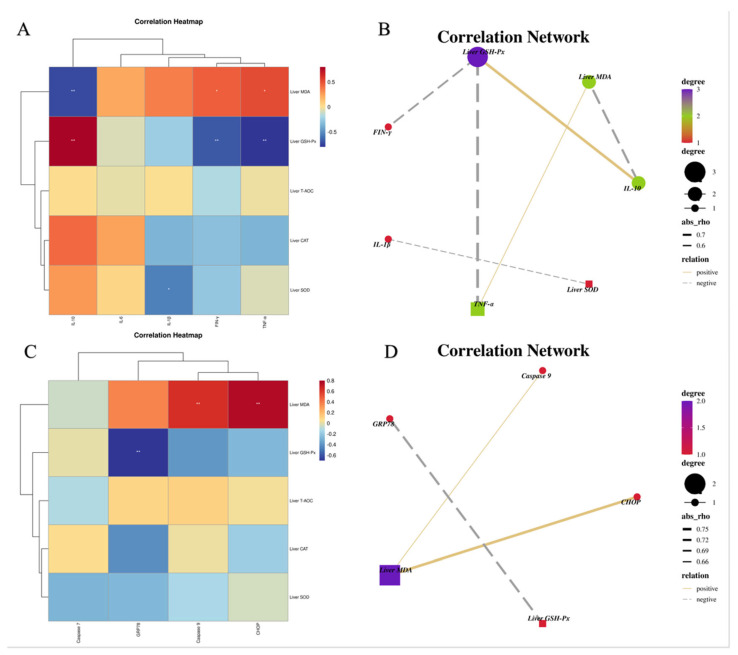
Correlation heatmap (**A**) and correlation network (**B**) between inflammatory cytokines and antioxidant indexes in the liver; correlation heatmap (**C**) and correlation network (**D**) between ERS parameters and antioxidant indexes in the ileum. * *p* < 0.05 and ** *p* < 0.01.

We next explored the potential correlation among inflammatory cytokines, ERS parameters and antioxidant indices in the liver (Figure 9). Liver GSH-Px was positively correlated with IL−10 and inversely correlated with TNF−α and FIN−γ. Likewise, liver MDA was positively correlated with TNF−α and negatively correlated with IL−10. Moreover, liver GSH−Px was negatively correlated with GRP78. Liver MDA was positively correlated with Caspase 9 and CHOP.

### 3.10. CSB Regulated the Nrf2 Antioxidant Signaling Pathway

**Figure 10:**
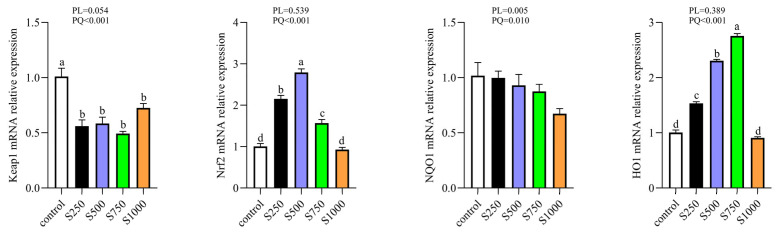
Effects of CSB supplementation on Nrf2 signaling-related gene expression in the liver. PL: *p*-value for linear effect. PQ: *p*-value for quadratic effect. ^a–d^ Means with unlike letters are significantly different (Turkey, *p* < 0.05).

Figure 10 displays the mRNA levels of Nrf2 and its downstream target gene. The mRNA levels of Nrf2 and HO1 markedly increased in a quadratic manner with an increase in CSB supplementation (*p* < 0.05). However, dietary CSB treatment markedly downregulated the mRNA expression of Keap1 in a quadratic manner (*p* < 0.05).

## 4. Discussion

Oxidative stress occurs when the oxidation resistance of cells and the extracellular space is overpowered by exogenous or endogenous reactive oxygen species [25]. Hens, especially in the late stage of laying, are more vulnerable to sustaining oxidative stress damage due to long-term egg production and body metabolism associated with the generation of massive radical substances and active oxygen species [26]. The antioxidant enzyme system, such as GSH-Px, CAT, T-AOC and SOD, plays a vital role in scavenging free radicals and sustaining the intracellular redox equilibrium [27]. One of the important mechanisms by which CSB supplementation attenuates oxidative stress damage may involve an enhanced antioxidant status. In the current study, apart from increasing GSH-Px and T-AOC activities, the MDA content was diminished quadratically by CSB supplementation in the serum and liver. A similar finding was reported that the administration of sodium butyrate to the diets of dairy goats enhances antioxidant stability in sub-acute ruminal acidosis [28]. MDA level is routinely applied as an index to evaluate lipid peroxidation [29]. Furthermore, previous studies have also demonstrated that treatment with sodium butyrate could restore antioxidant capacity and lower the MDA concentration to prevent lipid peroxidation in the liver of rats fed with a high-fat diet [30]. The integrated results indicate that supplementing the hen diet with CSB boosts antioxidant activity and prevents lipid peroxidation, thereby lowering oxidative stress in the body.

In avian species, the liver is the main site of lipogenesis, accounting for about 95% of de novo fatty acid synthesis [31]. Due to the exceptional genetic selection for the laying property of hens, the requirement for lipid oxidation and metabolism in vivo is exuberant, leading to generous lipid deposition in the liver during the middle and later stages of laying [32]. Previous studies have mainly focused on the alleviating effects of sodium butyrate on lipid deposition in swine or rats [33]. So far, as we know, our current study is the first to demonstrate the effects of CSB supplementation on the hepatic lipid metabolism in laying hens. In this study, the diet with CSB supplementation reduced lipid droplet deposition in the S500 and S750 groups and then increased with the successive increase in CSB addition in post-peak laying hens, as demonstrated by the results of Oil Red O staining, which revealed that CSB is beneficial to ameliorate lipid deposition at a reasonable dose. The hallmark of hepatic steatosis is the abnormal accumulation of TG and NEFA, which may lead to lipid peroxidation and additional histological changes [4]. It has been reported that dairy cows with fatty livers exhibited high levels of TG and NEFA [34]. The levels of HDL-C and LDL-C are associated with chronic maladies incidence, such as NAFLD and CVDs [35]. The current study found that CSB decreased the content of TG, LDL-C and NEFA, and increased the HDL-C level in the liver, which verified that CSB is conducive to preventing lipid accumulation and peroxidation and chronic diseases.

To elucidate the underlying mechanism of the repression effect of CSB on lipid deposition in laying hens, we further analyzed the gene expression by adjusting the hepatic fat metabolism of laying hens. The sustenance of the intracellular lipid steady state mainly relies on the dynamic equilibrium between lipid catabolism and biosynthesis [36]. An enormous amount of evidence has suggested that butyrate could ameliorate hepatic steatosis by reducing adipogenesis or enhancing intracellular lipolysis activity [13,37]. Our results showed a remarkable decrease in the hepatic mRNA levels of FASN and ACC and a dramatic increase in the hepatic mRNA expression of PPARα and CPT1. In the liver, ACC, which could catalyze the conversion of acetyl-CoA to malonyl-CoA, is the rate-limiting enzyme in the fatty acid synthesis step [38]. Moreover, it has been reported that FASN is involved in fatty acid biosynthesis, and the suppression of FASN activity could lessen adiposeness [39]. Nevertheless, PPARα and CPT-I are pivotal genes in commanding mitochondrial, beta-oxidation of fatty acids and peroxisomal fatty acid oxidation [40,41,42]. In view of the foregoing, CSB expedited lipid metabolism, possibly by inhibiting hepatic lipogenesis and promoting hepatic lipolysis. Additionally, the Pearson correlation analysis showed that liver GSH-Px and SOD activities were positively correlated with lipolysis-related genes and inversely associated with lipid synthesis-related genes, as well as reverse evidence of the MDA effect. Hence, we suggest that the improved lipid metabolism with dietary CSB addition in this study could be ascribed to elevated oxidative stability.

It has been reported that luxuriant deposition of lipids can induce lipocytes to exude massive pro-inflammatory cytokines and trigger inflammation, further aggravating metabolic disturbance in the body [43,44]. Our results indicated that CSB is conducive to relieving lipid deposition in the liver. To probe the underlying mechanism of the CSB addition reducing hepatic lipid accumulation, H&E staining involved in the liver pathological status was further assayed and demonstrated that the liver fat vacuole in CSB intervention was clearly lower, the arrangement of hepatic cells returned to normal, and the infiltration of inflammatory cells was reduced, which is consistent with the results of lipid metabolism. After that, we detected the cytokine mRNA levels in the liver and showed that the TNF-α and IFN-γ mRNA levels were downregulated and IL-10 mRNA levels were upregulated in the CSB treatment group, which is in accordance with the observation of HE staining. IFN-γ secreted by Th1 cells and TNF-α produced by macrophages have been considered cytokine mediators of local and systemic inflammation [45,46,47], which is an essential part of the immune inflammatory response that provokes the release of multiple inflammatory factors. In addition, IL-10, secreted by M2 macrophages, is a pleiotropic anti-inflammatory cytokine that blocks the production of pro-inflammatory cytokines [48,49]. Several studies have demonstrated that butyrate has an anti-inflammatory capacity as well as a latent capacity to irritate the immune system [50,51]. Therefore, the possible reason CSB may improve liver morphology is that CSB activated the inflammatory defense system.

Endoplasmic reticulum stress is linked to the induction of inflammation and oxidative stress [52]. Unfolded or misfolded proteins head up in the endoplasmic reticulum, which causes ERS. The glucose-regulated protein GRP78 and ERS biomarker CHOP are specific transcription factors for the procedure of ERS [53]. In the ERS pathway, GRP78 is isolated from transmembrane signaling protein, resulting in the activation of the apoptotic proteins (CHOP and caspase-12) and facilitating apoptosis [54]. Our data revealed that CSB decreased the ER stress-related index levels, such as GRP78, CHOP and caspase 9, suggesting that CSB reduced ERS and suppressed hepatic cell apoptosis. Similar studies were confirmed by Hu et al. [55], who showed that sodium butyrate improved insulin resistance by preventing ERS and protecting islet cells from apoptosis in type 2 diabetic rats. In addition, the relationships between inflammatory cytokines, ERS and antioxidant indexes were determined using Pearson correlation analysis. Thus, we speculated that the anti-inflammatory and anti-stress influence of sodium butyrate may be associated with the enhancement of antioxidant properties, but the concrete mechanism of oxidation resistance supporting these associations needs to be further explored.

In view of the correlation between the lipid metabolism-related enzymes, inflammatory cytokines, endoplasmic reticulum stress indexes and antioxidant parameters, we investigated the underlying molecular mechanisms of the effect of dietary CSB supplementation on the elevated antioxidative ability of laying hens. Nrf2 plays a vital part in adjusting antioxidant enzymes, thereby ameliorating oxidative damage. Emerging evidence indicated that sodium butyrate defends HepG2 cells from oxidative stress by regulating the Nrf2 signaling pathway [56]. Congruously, we demonstrated that dietary CSB addition upregulated the gene expression of Nrf2. Next, we evaluated the downstream and upstream molecules of the Nrf2 signaling way. Keap1 is an inhibitor of Nrf2 and can bind to Nrf2 to form Nrf2-Keap1, which inhibits the protective effect of Nrf2 on antioxidant genes [57,58]. HO1 and NQO1 are well-known downstream factors of the Nrf2 signaling pathway, which can scavenge free radicals to protect cells from oxidative stress [59]. As expected, our results showed that dietary CSB addition downregulated the Keap1 gene expression but upregulated the HO1 and NQO1 gene levels in laying hens. Taken together, the above results indicated that CSB strengthened the hepatic antioxidant property of laying hens, possibly attained by the Nrf2 signaling pathway.

## 5. Conclusions

**Figure 11:**
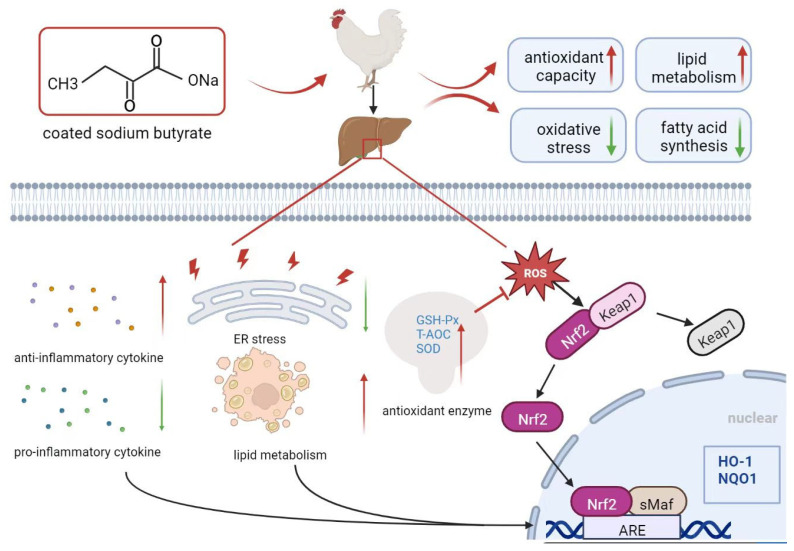
Graphical summary of dietary CSB supplementation to protect against liver damage and improve lipid metabolism in post-peaking laying hens by enhancing hepatic antioxidative function.

In conclusion, dietary CSB supplementation exerted a beneficial effect on the hepatic health status and alleviated oxidative stress in post-peak laying hens. In particular, CSB administration reduced the formation of lipid droplets, hepatic steatosis, and hepatic lipid deposition by enhancing the antioxidant capacity, reducing the inflammatory response and suppressing fatty acid synthesis. Moreover, the CSB used in this study is a feed additive with potential benefits in protecting against liver injury by modulating the Nrf2 antioxidant signaling pathway (Figure 11).

## Figures and Tables

**Table 1:** Ingredient compositions and nutrient levels in basal diet of hens.

Items	Composition
Ingredients	Content (%)
Corn	62
Soybean meal	24.5
Soybean oil	0.5
Limestone Premix ^1,2^	8 5
Total	100
Nutrient ^3^	
Metabolism energy, MJ/kg	10.99
Crude protein, %	15.67
Lysine, %	0.80
Methionine, %	0.34
Calcium, % Total phosphorus, %	3.69 0.54

^1^ The premix provided following per kilogram of diet: vitamin A, 7500 IU; vitamin D3, 2500 IU; vitamin E, 49.5 mg; vitamin K3, 2.5 mg; vitamin B1, 1.5 mg; vitamin B2, 4 mg; vitamin B6, 2 mg; vitamin B12, 0.02 mg; niacin, 30 mg; folic acid, 1.1 mg; pantothenic acid, 10 mg; biotin, 0.16 mg; chloride choline, 400 mg; sodium chloride, 2500 mg; Fe, 80 mg; Cu, 20 mg; Mn, 60 mg; Zn, 80 mg; I, 0.8 mg; Se, 0.3 mg. ^2^ The premix in 6 treatments provided per kilogram of diet: sodium butyrate, 250, 500, 750 and 1000 mg, respectively, and in the control without additional sodium butyrate. ^3^ Values were calculated from the Chinese feed database provided with tables of feed composition and nutritive values in China (21st edition).

**Table 2:** Primer used for real-time quantitative fluorescence PCR analysis.

Target Gene	Primer	Primer Sequence (5′-3′)	Accession No.
β-Actin	Forward	TCCCTGGAGAAGAGCTATGAA	NM_205518.1
	Reverse	CAGGACTCCATACCCAAGAAAG	
SREBP-1c	Forward	GCCATCGAGTACATCCGCTT	NM_204126.2
	Reverse	GGTCCTTGAGGGACTTGCTC	
FASN	Forward	GAATCCAGAAGGGCCAACGA	NM_205155.4
	Reverse	TCCAAGGGAGCAGCTTTTGT	
ACC	Forward	TACAGAGGTACCGGAGTGGT	NM_205505.1
	Reverse	TCTTCCCGAAGGGCAAAGAC	
PPARα	Forward	AGGCCAAGTTGAAAGCAGAA	NM_001001464.1
	Reverse	TTTCCCTGCAAGGATGACTC	
CPT1	Forward	GGCTCTGGCAGGAGCTACA	XM_040700878.2
	Reverse	CACTGCAGCTGGGATCTTGA	
ACOX1	Forward	ACTGAGCTGTGTCTCTTGTATG	XM_015295164.2
	Reverse	GCTTCAGGTGTTTGTGGAAAG	
IFN-γ	Forward	AGCTGACGGTGGACCTATTATT	NM_205149.1
	Reverse	GGCTTTGCGCTGGATTC	
TNF-α	Forward	GACAGCCTATGCCAACAAGTA	AY765397.1
	Reverse	TCCACATCTTTCAGAGCATCAA	
IL6	Forward	CTCGTCCGGAACAACCTCAA	NM_204628.2
	Reverse	TCAGGCATTTCTCCTCGTCG	
IL10	Forward	CCAGGGACGATGAACTTAACA	NM_001004414.2
	Reverse	GATGGCTTTGCTCCTCTTCT	
IL1β	Forward	ACTGGGCATCAAGGGCTA	XM_015297469.2
	Reverse	GGTAGAAGATGAAGCGGGTC	
GRP78	Forward	GTTACTGTGCCAGCCTACTT	NM_205491.1
	Reverse	CCGCTTCGCTTTCTCTACTT	
Caspase 9	Forward	GACCTGCTAACCATGCTACTT	XM_424580.6
	Reverse	TTCCACTGAATCCTCCAATCC	
Caspase 7	Forward	TGCAAAGCCAGACAGAAGTAG	XM_025151846.1
	Reverse	GGTCCATCGGTGCCATAAAT	
CHOP	Forward	GCACAGCCCATTTCTGTTTC	XM_015273173.2
	Reverse	TGCCATCCCATTCTGCTAAG	
Keap1	Forward	TGCCCCTGTGGTCAAAGTG	XM_015274015.1
	Reverse	GGTTCGGTTACCGTCCTGC	
Nrf2	Forward	TGTGTGTGATTCAACCCGACT	NM_205117.1
	Reverse	TTAATGGAAGCCGCACCACT	
HO1	Forward	TTGGCAAGAAGCATCCAGA	NM_205344.1
	Reverse	TCCATCTCAAGGGCATTCA	
NQO1	Forward	AAAGCCATGCTGTCACTCACC	NM_001277619.1
	Reverse	GTAGCGCACGCTGTAGCAAAT	

## Data Availability

Data is contained within the article.
